# Microbial communities and their association with soil health indicators under row cash crop and cover crop diversification: a case study

**DOI:** 10.3389/fmicb.2025.1664417

**Published:** 2025-09-29

**Authors:** Rachel Wooliver, Stephanie N. Kivlin, Sindhu Jagadamma

**Affiliations:** ^1^Department of Biosystems Engineering and Soil Science, University of Tennessee, Knoxville, TN, United States; ^2^Department of Ecology and Evolutionary Biology, University of Tennessee, Knoxville, TN, United States

**Keywords:** arbuscular mycorrhizal fungi, cover crop, crop diversification, plant pathogen, rotational diversity, row crop, soil microbial community, soil health

## Abstract

**Introduction:**

Crop diversification is an emerging approach for increasing soil health and agroecosystem sustainability. By diversifying residue inputs to soils, plant diversity can increase microbial community diversity and function, foster arbuscular mycorrhizal fungal (AMF) relationships, and limit plant vulnerability to pathogens. However, crop diversification approaches are usually limited in plant species diversity, for example adding one or two species as a cover crop or cash crops in rotation.

**Methods:**

We implemented a four-year field experiment combining two crop diversification strategies (cover cropping and crop rotation) on a silt loam soil in western Tennessee, USA, to determine influences on soil microbial community diversity and composition, and their association with soil health indicators. Treatments ranged from simplified continuous corn (*Zea mays* L.) or soybean (*Glycine max* L.) with winter fallow to a three-species [corn-cotton (*Gossypium hirsutum* L.)-soybean] annual crop rotation with a five-species winter cover crop mix. We characterized bacterial and fungal communities at three timepoints per year (spring, fall, summer) and quantified a suite of soil health indicators at each timepoint.

**Results:**

Microbial diversity did not increase with crop diversity. However, bacterial community composition responded to crop rotation treatments differently across timepoints, and fungal community composition responded to cover crop and crop rotation treatments differently across timepoints. For example, the five-species cover crop mix increased relative abundance of AMF (Glomeromycota) in the first year, and crop rotations reduced the relative abundances of fungal plant pathogens found in continuous soybean (*Plectosphaerella*, *Paraphoma*, and *Fusariella*) and continuous corn (Didymellaceae) in multiple years. Microbial community composition was strongly linked to all soil health indicators, especially moisture content, phosphatase activity, β-glucosidase activity, water-extractable organic carbon, and nitrate-nitrogen, despite minimal effects of crop diversification on soil health indicators.

**Discussion:**

We conclude that 4 years of crop rotation and diverse cover crops have strong but separate and season-dependent potentials to decrease fungal pathogens and increase AMF abundance, respectively. However, linkages between microbial communities and soil health are largely independent of crop diversification.

## 1 Introduction

Microbial communities, as the engines of soil functioning (Graham et al., 2016), are central to soil health and food production (Bertola et al., 2021; Gayan et al., 2023; Wei et al., 2024). Soil microbial community composition and diversity are shaped by several factors, including plant communities, with higher supply of more diverse plant inputs to soil leading to more active and diversified soil microbial communities and their functioning (Zak et al., 2003; Cline et al., 2018; Chen et al., 2019; Prommer et al., 2020). In agroecosystems, there is growing evidence that diversification of crop species improves nutrient cycling, soil organic carbon (C) storage, and crop yield (Iverson et al., 2014; Tamburini et al., 2020; Garland et al., 2021). As a result, diverse soil microbial communities drive greater soil multifunctionality, with higher capacity to decompose organic matter, cycle key nutrients for plant growth, and maintain soil functioning under stressful conditions (Griffiths and Philippot, 2013; Delgado-Baquerizo et al., 2020; Garland et al., 2021). In turn, plants perform better in soils with higher microbial diversity (Heijden et al., 2008; Li et al., 2025). However, crop productivity around the globe is increasingly threatened by multiple factors, including drought (Kuwayama et al., 2019) and proliferation of pests and pathogens (Savary et al., 2019). It remains uncertain whether crop diversification strategies can be used to alleviate these pressures by altering soil microbial communities.

Crop diversification is one of several agricultural management practices recommended for enhancing agroecosystem functioning globally. However, outcomes of crop diversification for soil biology and function are less well-represented compared to soil chemical changes such as nutrient cycling and overall crop productivity (Tamburini et al., 2020). Moreover, benefits may be underestimated if a limited number of species is employed. Common crop diversification strategies include cover cropping (i.e., introduction of a non-yielding crop during what would otherwise be a fallow period) and crop rotation (i.e., planting different cash crop species over time). Typically, only one or two species are added to cropping systems through crop diversification, such as a single species of grass or legume added as a cover crop (Jian et al., 2020). Few studies highlight the potential for the addition of multiple plant species to a cropping system to enhance soil microbial diversity and functional benefits beyond that of adding one crop species alone. For example, a study on intercropping showed that four-species crop mixtures increased soil microbial diversity by 35% relative to crop monoculture, compared to a 15% increase from a two-species mixture (Stefan et al., 2021). Additionally, evidence from natural ecosystems (e.g., forests and alpine systems) suggests that soil microbial diversity and functioning continue to increase with plant species diversity beyond the addition of only a few species (Porazinska et al., 2018; Hanif et al., 2019).

Because different soil microbes are important for specific functions in agricultural soils, outcomes of crop diversification may depend on changes in microbial community composition in addition to diversity. Among the most impactful on crop performance and soil health are arbuscular mycorrhizal fungi (AMF) and disease-causing pathogens (Chernov and Semenov, 2021). Constituting a majority of the phylum Glomeromycota, AMF are obligate mutualists that contribute to crop nutrient and water acquisition, growth, and tolerance to abiotic and biotic stressors (Wahab et al., 2023). Cover crops and crop rotation can foster higher abundances of AMF, and this is one pathway through which crop diversification increases main crop nutrition and yield (Deguchi et al., 2007; Fang et al., 2021; Guzman et al., 2021). AMF communities also play a key role in soil C cycling, contributing to both soil organic matter decomposition and the formation and stabilization of soil organic C (Wei et al., 2019; Conti et al., 2025). According to a global meta-analysis, inoculating grain and non-grain crops with AMF spores increases soil organic C by an average of 21.5% (Conti et al., 2025) and crop yield by an average of 23% (Wu et al., 2022), highlighting their centrality to agroecosystem functioning. Pathogens also strongly affect crop performance, together with pests contributing to yield losses of 22.5% on average across wheat (*Triticum aestivum* L.), rice (*Oryza sativa* L.), corn (*Zea mays* L.), potato (*Solanum tuberosum* L.), and soybean (*Glycine max* L.) worldwide (Savary et al., 2019). A grassland experiment showed that higher plant diversity reduces plant infection rate (Rottstock et al., 2014). In row cropping systems, crop rotation is used to reduce diseases with a limited host range, as the pathogen does not survive on the rotated crop (Liu et al., 2022). For example, Gahagan et al. (2023) showed that corn-soybean-wheat rotations had lower abundances of pathogenic species within the fungal genera *Fusarium*, *Globisporangium*, and *Saprolegnia* relative to monoculture wheat. While some cover crops decrease the susceptibility of subsequent crops to pathogens, others can increase it, particularly those that cause root rot such as *Fusarium* (Ray et al., 2022). In the latter case, cover crops may be acting as a “green bridge,” or a living host that allows the transfer of a pest or disease across crop cycles (Acharya et al., 2017). Thus, the influence of crop diversification on soil microbial communities and soil functioning may depend on crop species capacities to serve as a host for different members of the microbial community.

To explore outcomes of crop diversification for soil microbes and soil functioning, we conducted a four-year agricultural field experiment in western Tennessee, United States. This is an important area for corn, cotton (*Gossypium hirsutum* L.), and soybean production (McClure, 2009, 2022; Main, 2012), however the accumulation of soil organic matter remains a major challenge due to nutrient-limited, highly erodible soils and warm, humid climate. The field experiment employed a factorial treatment design including two crop diversification strategies, winter cover cropping and annual row crop rotation. We used amplicon sequencing to characterize fungal and bacterial communities three times per year for 4 years. We also quantified a panel of soil health-related properties. We hypothesized that (1) the most diverse cropping systems would have the highest soil microbial community diversity, the highest abundances of AMF, and the lowest abundances of plant pathogens, and that (2) changes in soil microbial communities would be associated with biological, physical, and chemical soil health indicators.

## 2 Materials and methods

### 2.1 Field experiment and soil collection

We initiated a crop diversification field experiment in September of 2020 at the University of Tennessee’s AgResearch and Education Center in Jackson, TN, USA (35° 37′59.4″ N, 88° 51′36.4″ W). At the site, historical mean annual temperature and precipitation were 15.74 °C and 1,437 mm, respectively, and the soil was a Lexington silt loam (fine-silty, mixed, thermic Ultic Hapludalfs). Soybean, wheat, and corn were grown at the site in 2017, 2018, and 2019, respectively. At the beginning of the experiment, the surface soil (0–10 cm) had 6.64 pH, 1.32 g cm^−3^ bulk density, 9.41 g kg^−1^ organic C, 1 g kg^−1^ total N, 22% sand, 62% silt, and 16% clay. The experimental design was a split-plot randomized complete block design, with 80 plots total (four crop rotation treatments × five cover crop treatments × four blocks). Annual crop rotation treatments (main plots) included continuous corn, continuous soybean, corn-soybean rotation, and corn-cotton-soybean rotation. Winter cover crop treatments (split-plots) included a no cover crop control (winter fallow), monoculture crimson clover (*Trifolium incarnatum* L.), monoculture winter wheat, a two-species mixture of clover and wheat, and a five-species mixture of clover, cereal rye (*Secale cereale* L.), oat (*Avena sativa* L.), clover, hairy vetch (*Vicia villosa* Roth), and Daikon radish (*Raphanus sativus* L.). Plots were maintained as no-till and irrigated only when necessary to ease drought stress. Cover crops were drilled on 19-cm rows each October–November and terminated with general-use herbicides each March–April. Cash crops were planted at least 2 weeks after cover crop termination on 76.2-cm rows and received fertilizers as recommended by the University of Tennessee Crop Production Programs (Duncan et al., 2015).

We collected soil samples at three timepoints per year, including the spring (immediately prior to cash crop planting in April–May), summer (during the active growth of cash crops in July–August), and fall (after cash crop harvest in September–October). Sampling occurred from fall 2020 (baseline, prior to planting cover crops) through fall 2024 (after 4 years of treatments). In total, there were 1,040 soil samples (13 timepoints × 80 plots). From each split-plot, we collected 6–10 samples to a depth of 10 cm using stainless steel probes (2.5-cm-diameter). Samples were composited in plastic zip-top bags and stored at 4 °C during transport to the laboratory (24–48 h), where soils were homogenized and cleared of visible residues. Subsamples were taken to quantify a suite of properties considered early indicators of changes in soil health, including moisture content, microbial biomass C, potential enzyme activities, nitrate- and ammonium-N concentrations, and water-extractable organic C (Stott, 2019). Fresh subsamples were stored at 4 °C for less than 1 week prior to estimation of gravimetric moisture content and microbial biomass C. An additional two subsamples were frozen (−20 °C) for up to 2 months prior to microbial community and enzyme analyses. The rest of the soil was air-dried for 2 weeks and passed through a 2-mm sieve, subsamples of which were used to determine inorganic N and water-extractable organic C concentrations.

### 2.2 Soil microbial community characterization

We extracted soil DNA using PowerSoil Pro DNA Isolation Kits (MoBio Laboratories, Inc.) following company protocol, except that ATL buffer (MoBio Laboratories, Inc.) was added at the lysis stage to increase DNA yield. Total DNA was quantified with a Nanodrop spectrophotometer (Thermo Scientific) and diluted to equal concentrations using PCR-grade water. Bacterial and fungal libraries were prepared and sequenced at the University of Tennessee’s Illumina Sequencing facility using primers targeting the V4 region of 16S rRNA (341F/785R) genes (Klindworth et al., 2013) and fungal ITS (ITS4fun/5.8Sfun) genes (Cregger et al., 2018). Sequencing was performed using MiSeq (fall 2020 through spring 2023 samples) or NextSeq (summer 2023 through fall 2024 samples) platforms. We did not expect communities to differ between sequencing platforms, as MiSeq and NextSeq yield nearly identical community compositions (Walsh et al., 2018). All samples were trimmed to the same read length (2 × 275 nucleotides). We used the R-based DADA2 pipeline (Callahan et al., 2016) to filter and group sequences into amplicon sequence variants (ASVs), then to assign taxonomy to each ASV based on ITS UNITE (Nilsson et al., 2019) and 16S RDP (Cole et al., 2014) databases. All samples were rarefied to 5,000 reads using the R package *vegan* (Oksanen et al., 2013) to account for variation in sequencing depth across samples, resulting in the exclusion of 58 bacterial samples and 19 fungal samples. This number was chosen to minimize data loss while maximizing sampling depth, which ranged from 43 to 553,035 reads for bacteria and zero to 173,720 reads for fungi. We calculated diversity indices, including richness, Shannon’s index, Simpson’s index, and Pielou’s evenness, using the R packages *vegan* (Oksanen et al., 2013) and *microbiome* (Pielou, 1966; Lahti and Shetty, 2019). Raw sequences are available in NCBI SRA under BioProject ID PRJNA1265188.

### 2.3 Soil health indices

Gravimetric moisture content (g moisture g dry soil^−1^) was measured by drying a 10-g portion of fresh soil at 105 °C. Microbial biomass C (mg C kg dry soil^−1^) was determined using the chloroform fumigation method (Vance et al., 1987) updated by Scott-Denton et al. (2006). In short, 5–10 g of fresh soil was extracted with 0.5 M K_2_SO_4_, centrifuged for 3 min at 500 g, and filtered (Fisher Q5 filter paper). Simultaneously, another 5–10 g of fresh soil was fumigated for 24 h in a 50-mL centrifuge tube by adding 2 mL of ethanol-free chloroform onto a cotton ball positioned above but not touching the soil, ventilated, and extracted as above. Extracts were analyzed for organic C concentration using a colorimetric assay of Mn(III)-pyrophosphate oxidizable C (Bartlett and Ross, 1988). Microbial biomass C was calculated as the difference in C concentration between the fumigated and unfumigated extracts, divided by an extraction efficiency of 0.45 (Wu et al., 1990).

Potential activities of three extracellular hydrolytic enzymes were measured using fluorometric assays (Bell et al., 2013). The enzymes included one C-cycling enzyme (β-glucosidase), one N-cycling enzyme (N-acetyl-β-glucosaminidase), and one P-cycling enzyme (phosphatase). Briefly, soils were blended in a 50 mM sodium acetate buffer adjusted to a pH of 6.6 and transferred to a deep well plate, where they were combined with enzyme substrates tagged with a fluorescent marker (4-methylumbelliferone or 7-amino-4-methylcoumarin). In a different deep well plate, the blended soils were combined with a range of fluorescent marker standard concentrations. The plates were incubated at 25 °C for 3 h. After centrifuging, supernatants were transferred to black well plates and fluorescence was measured at wavelengths targeting each marker (BioTek Synergy HTX Multimode reader, Agilent, United States). Potential enzyme activity was calculated in units of nmol activity g dry soil^−1^ h^−1^ based on fluorescent marker standard curves. Each sample was assayed twice and averaged.

Nitrate- and ammonium-N concentrations (mg N kg dry soil^−1^) were determined using colorimetric assays (Verdouw et al., 1978; Forster, 1995; Doane and Horwáth, 2003) after soil extraction in 2 M KCl, centrifuging for 3 min at 500 g, and filtration (Fisher Q5 filter paper). Water-extractable organic C concentration (mg C kg dry soil^−1^) was determined using a vario TOC Cube CN analyzer (Elementar, Germany) after soil extraction in MilliQ water, centrifuging for 30 min at 1,096 g, and filtration (Fisher Q2 filter paper).

### 2.4 Statistical analysis

All analyses were implemented in R Statistical Software v4.4.3 (R Core Team, 2025), and R codes with data are available at https://github.com/rwoolive/usda-cdp-microbes. We determined treatment effects on total bacterial and fungal diversity using mixed-effects ANOVAs implemented with the *nlme* package (Pinheiro et al., 2022). Richness was chosen to represent microbial diversity because it more often met the statistical assumptions of normal residuals and equal group-level variances compared to the other indices, however we include the results for Shannon’s index, Simpson’s index, and Pielou’s evenness in Supplementary Table S1. Models included cover crop, crop rotation, timepoint, and all interactions as main effects, and block as a random effect. If we recovered significant interactive effects, additional models were implemented to explore variation in treatment effects within data subsets. Where necessary, response variables were log-transformed to improve residual normality. Fixed effects with *p* < 0.05 were considered significant.

We used non-metric multidimensional scaling (NMDS) and permutational analysis of variance (PERMANOVA) to explore microbial community turnover across cover crops, crop rotations, and timepoints using *vegan* (Oksanen et al., 2013). Similar to ANOVA models, if statistical interactions occurred, additional PERMANOVA models were implemented to determine how soil microbial community composition varied with treatments within data subsets. Based on PERMANOVA results, significant predictors of microbial communities were included as predictors in linear discriminant analysis effect size (LEfSE) analyses to identify microbial taxonomic groups whose abundances were associated with specific treatments (Segata et al., 2011). LEfSE analyses were implemented in the R package microbiomeMarker (Cao et al., 2022), using default settings, except *p*-value cutoff was set to 0.01 instead of 0.05, and linear discriminant analysis effect size cutoff was set to four instead of two, in order to reduce false positives. This approach yielded LEfSe scores representing how much more relatively abundant a taxonomic group is in a treatment compared to all other treatments. In this way, we were able to determine indicator taxa for specific treatments across multiple levels of taxonomic hierarchy (genus through phylum) without separately testing each taxon of interest. We used a fungal trait database to determine primary lifestyle (e.g., plant pathogen) of fungal indicator taxa (Põlme et al., 2020). Last, we used distance-based redundancy analysis (RDA) to determine which soil health indices were related to compositional changes in soil microbial communities. For RDA, stepwise model selection was used to identify the minimum set of soil properties that explained significant soil microbial community composition.

## 3 Results

### 3.1 Effects of crop diversification on soil bacterial communities

Across the rarefied dataset of 982 soil bacterial samples, we identified 34,603 ASVs within 29 phyla. Dominant phyla included Proteobacteria (26.09% of ASVs, which included 7.28% Alphaproteobacteria, 6.73% Betaproteobacteria, 7.75% Deltaproteobacteria, and 3.8% Gammaproteobacteria), Acidobacteria (17.13% of ASVs), Actinobacteria (13.16% of ASVs), and Planctomycetes (8.17% of ASVs). Bacterial diversity did not respond to cover crop or crop rotation treatments, but varied significantly across time (Table 1 and Supplementary Figures S1, S2).

**Table 1:** Three-way ANOVA of cover crop, crop rotation, and timepoint effects on soil microbial diversity.

Effect	Bacterial richness	Fungal richness
Cover	0.24	1.94
Rotation	0.59	1.04
Timepoint	6.88***	4.28***
Cover × rotation	1.15	1.03
Cover × timepoint	0.56	1.03
Rotation × timepoint	0.51	1.26
Cover × rotation × timepoint	0.65	0.60

Values represent *F*-values. Significant treatment effects at *p* < 0.001 are marked with ***.

**Figure 1:**
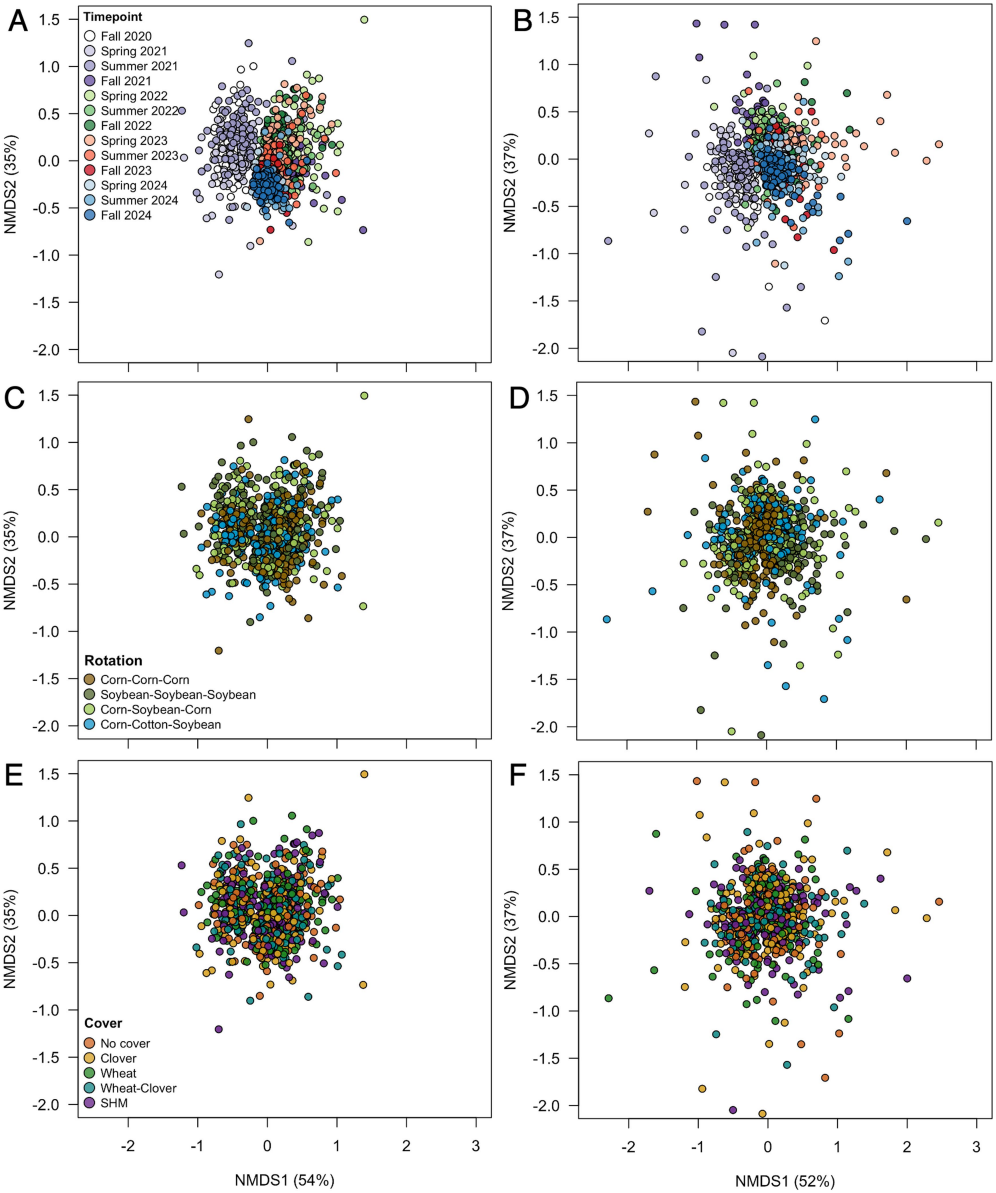
Microbial community composition across time for bacteria **(A,C,E)** and fungi **(B,D,F)** by timepoint **(A,B)**, rotation **(C,D)**, and cover crop **(E,F)**.

**Figure 2:**
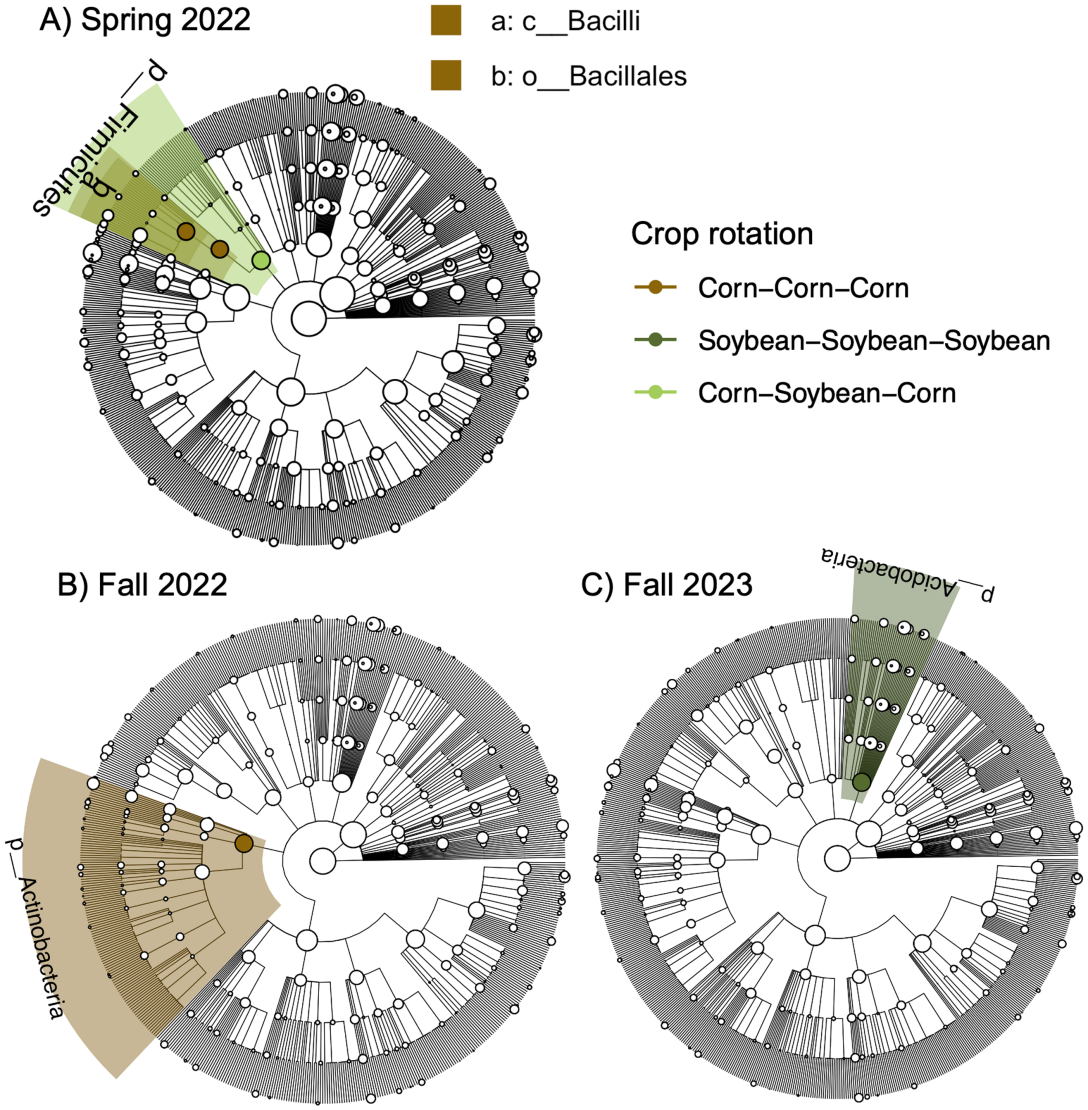
Bacterial indicator taxa for different crop rotation treatments across different sampling points based on linear discriminant analysis effect size (LEfSE) analysis.Sampling points include **A)** Spring 2022, **B)** Fall 2022, and **C)** Fall 2023. Taxa are mapped onto a 16S-based phylogeny.

Further, we identified a two-way interaction between crop rotation and timepoint for bacterial community composition (Table 2 and Figure 1). Additional models indicated that bacterial community composition responded significantly to crop rotation treatments at each timepoint (Table 3). Crop rotation explained increasing variation at each successive timepoint (4.3% in the summer of 2021 to 6.8% in the fall of 2024). There were four indicator taxonomic groups identified for crop rotation (Figure 2). In the spring of 2022, the phylum Firmicutes was an indicator taxonomic group for corn-soybean rotation, although only 1 year of corn had been implemented (in 2021). The class Bacilli (specifically, the order Bacillales) was an indicator taxonomic group for continuous corn, but again, only 1 year of corn had been implemented (in 2021). In the fall of 2022, after 2 years of corn, the phylum Actinobacteria was an indicator taxonomic group for continuous corn. In the fall of 2023, after three years of soybean, the phylum Acidobacteria was an indicator taxonomic group for continuous soybean. There were no indicator taxa identified in the remaining timepoints, or for the most diverse (corn-cotton-soybean) rotation.

**Table 2:** Total percent variance in soil microbial community composition explained by cover crop, rotation, timepoints, and all two- and three-way interactions, based on PERMANOVA.

Effect	Bacteria	Fungi
Cover	0.4*	0.8**
Rotation	1.2**	2.9**
Timepoint	12.7**	13.0**
Cover × rotation	1.2*	1.3**
Cover × timepoint	3.9	3.9
Rotation × timepoint	3.5**	5.3**
Cover × rotation × timepoint	11.2	10.1

Significant effects at *p* < 0.05 and *p* < 0.01 are marked with * and **, respectively.

**Table 3:** Total percent variance in soil microbial community composition explained by cover crop, rotation, and their interaction across different sampling points, based on PERMANOVA.

Microbial group	Effect	2021			2022			2023			2024		
		Spring	Summer	Fall	Spring	Summer	Fall	Spring	Summer	Fall	Spring	Summer	Fall
Bacteria	Cover	5.3	4.6	4.6	5.8	5.0	6.4	6.0	4.4	4.5	4.7	4.6	4.4
	Rotation	—	4.3**	5.3**	5.0*	5.4**	5.6**	5.2*	5.7**	5.8**	5.7**	6.3**	6.8**
	Cover*Rotation	—	13.8	13.9	15.7	14.3	17.7	17.60	13.2	12.7	13	13.8	12.8
Fungi	Cover	6.0**	5.0	4.7	6.2**	5.7**	5.2*	8.4**	5.0*	4.7	5.3*	5.1	4.4
	Rotation	—	5.7**	5.9**	7.6**	9.4**	12.8**	10.3**	12.5**	15.4**	11.7**	12.0**	13.3**
	Cover*Rotation	—	13.9	13.8	13.6	12.5	12.4	14.8	12.0	11.7	12.3	12.5	12.5

Significant effects at *p* < 0.05 and *p* < 0.01 are marked with * and **, respectively.

Bacterial community composition also showed a two-way interaction between crop rotation and cover crop (Table 2). Additional models indicated that cover crop explained 1.6–1.8% variation (*p* = 0.002) in composition within each crop rotation, and crop rotation explained 2.2–2.6% variation (*p* = 0.002) in composition within each cover crop. However, LEfSE analysis yielded no indicator taxa for any cover crop treatment within crop rotations, nor for any crop rotation within cover crops.

### 3.2 Effects of crop diversification on soil fungal communities

Across the rarefied dataset of 1,021 soil fungal samples, we identified 11,866 fungal ASVs within 18 phyla. Dominant phyla included Ascomycota (41.02% of ASVs), Chytridiomycota (16.44% of ASVs), Basidiomycota (14.17% of ASVs), and Glomeromycota (9.07% of ASVs). Similar to bacterial communities, fungal diversity did not respond to cover crop or crop rotation treatments, but varied significantly across time (Table 1 and Supplementary Figure S1).

**Figure 3:**
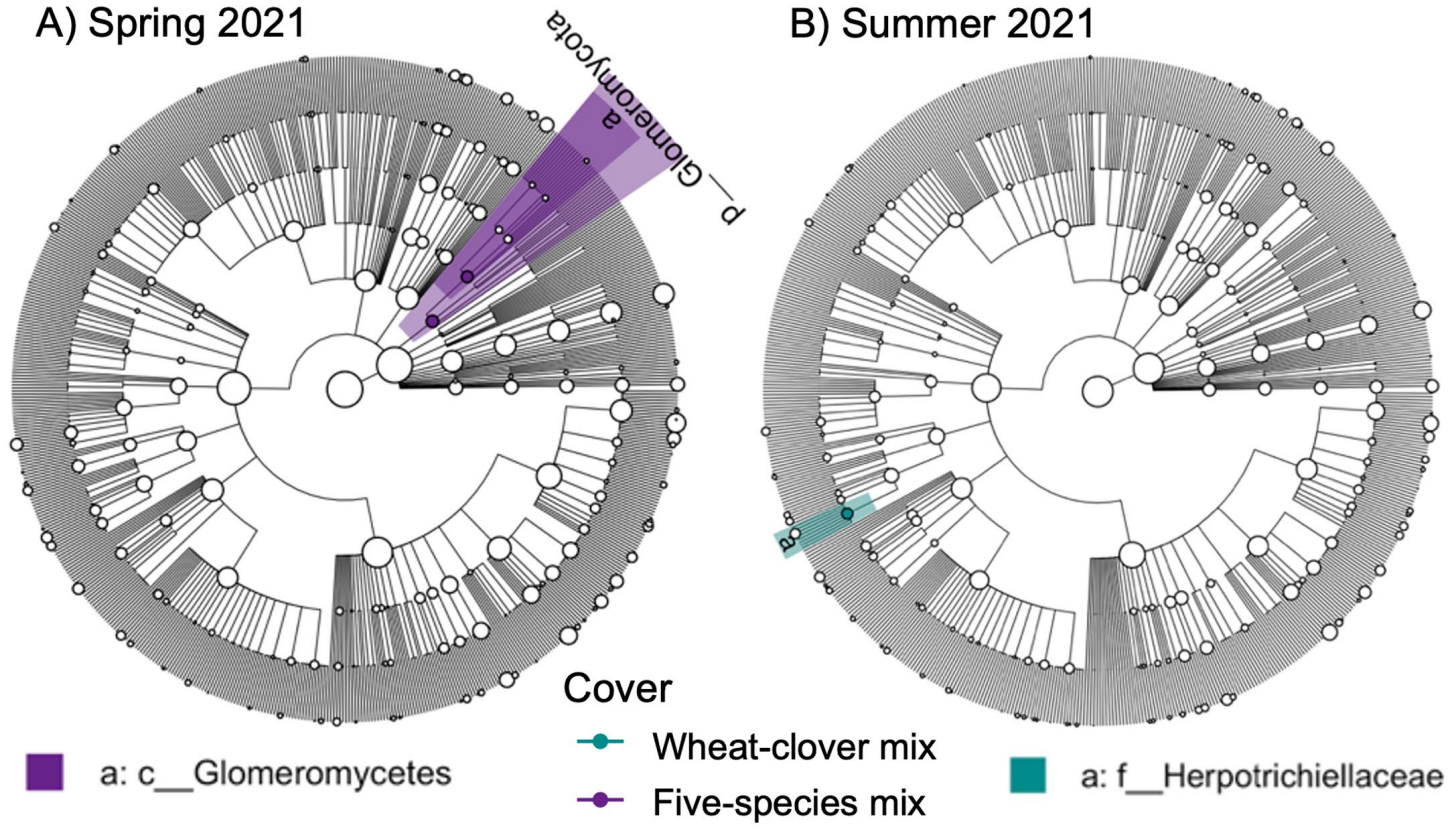
Fungal indicator taxa for different cover crop treatments at **A)** Spring 2021 and **B)** Summer 2021 sampling points based on linear discriminant analysis effect size (LEfSE) analysis. Taxa are mapped onto an ITS-based phylogeny.

**Figure 4:**
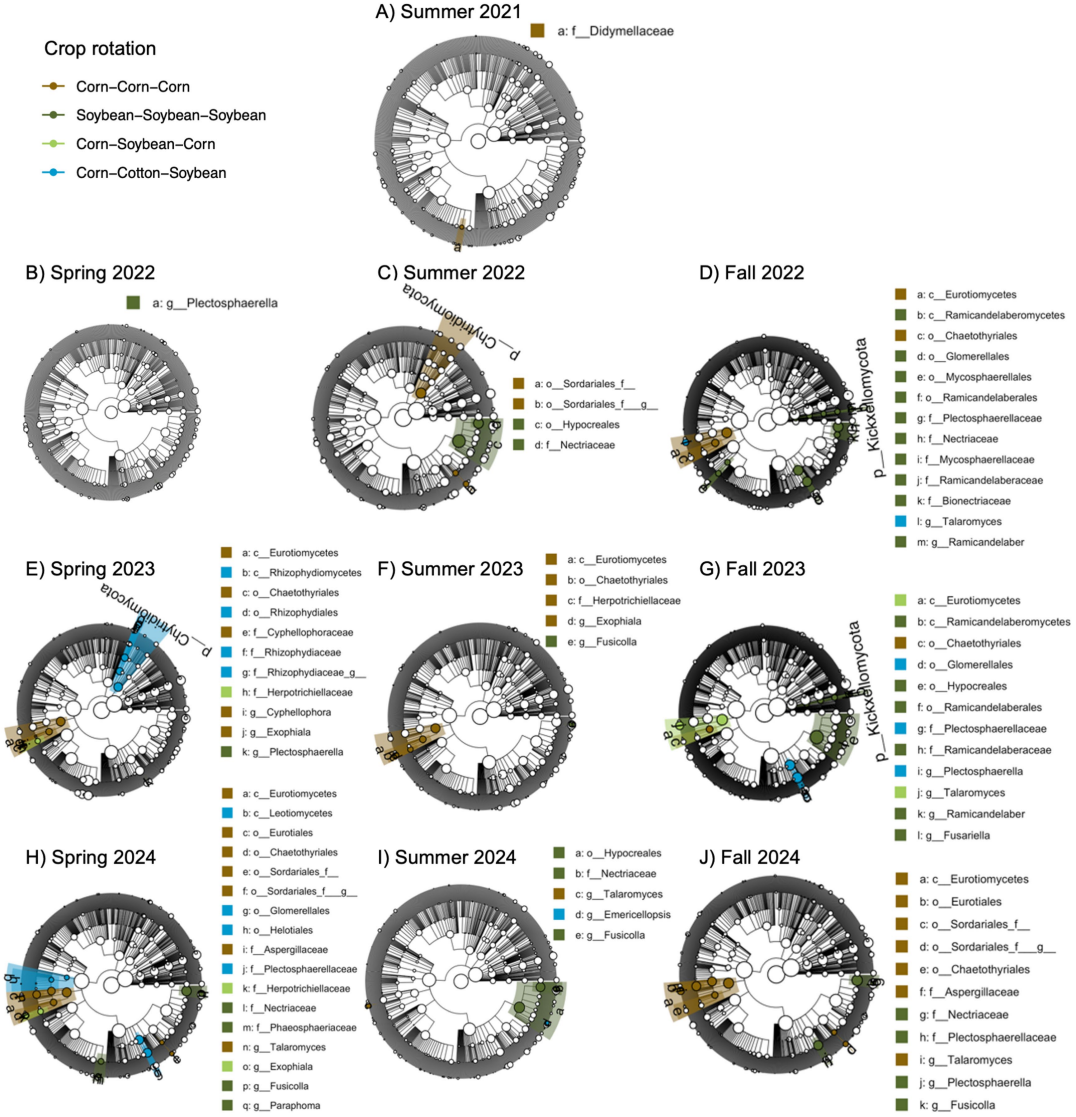
Fungal indicator taxa for different cropping system treatments across different sampling points based on linear discriminant analysis effect size (LEfSE) analysis. Sampling points include summer (A, C, F, I), spring (B, E, H), and fall (D, G, J), for years 2021 **(A)**, 2022 **(B-D)**, 2023 **(E-G)**, and 2024 **(H-J)**. Taxa are mapped onto an ITS-based phylogeny.

We also identified a two-way interaction between crop rotation and timepoint (Table 2 and Figure 1). Fungal community composition responded significantly to crop rotation treatment in all timepoints and to cover crop treatment in seven out of 12 timepoints (Table 3). Similar to bacterial communities, crop rotation tended to explain increasing variation in fungal communities across time (5.7% in the summer of 2021 to 15.4% in the fall of 2023). Cover crop explained the highest variation in fungal communities in the spring of 2023 (8.4%) (Table 3). There were two indicator taxonomic groups identified for cover crop treatment (Figure 3). In the spring of 2021, the phylum Glomeromycota (in particular, the class Glomeromycetes) was an indicator taxonomic group for the five-species cover crop mix. In the summer of 2021, the family Herpotrichiellaceae (a diverse family containing saprotrophs, plant pathogens, animal parasites, and lichenized fungi) was an indicator taxonomic group for the wheat-clover cover crop mix. No other timepoints showed fungal indicator taxa for cover crop treatments. However, there was a growing number of fungal indicator taxa identified for crop rotation treatments through time (Figure 4), including the family Didymellaceae (contains the plant pathogenic genus *Didymella*; continuous corn in summer 2021), and the genera *Plectosphaerella* (plant pathogen; continuous soybean in spring 2022, spring 2023, and fall 2024; corn-cotton-soybean rotation in fall 2023), *Fusariella* (plant pathogen; continuous soybean in fall 2023), *Paraphoma* (plant pathogen; continuous soybean in spring 2024), *Ramicandelaber* (saprotroph; continuous soybean in fall 2022 & 2023), *Talaromyces* (saprotroph; corn-soybean rotation in fall 2023 and continuous corn in spring, summer & fall 2024), *Emericellopsis* (saprotroph; corn-cotton-soybean rotation in summer 2024), *Fusicolla* (mycoparasite; continuous soybean in summer 2023 and spring, summer & fall 2024), and *Exophiala* (animal parasite; continuous corn in summer 2023 and spring 2024).

**Figure 5:**
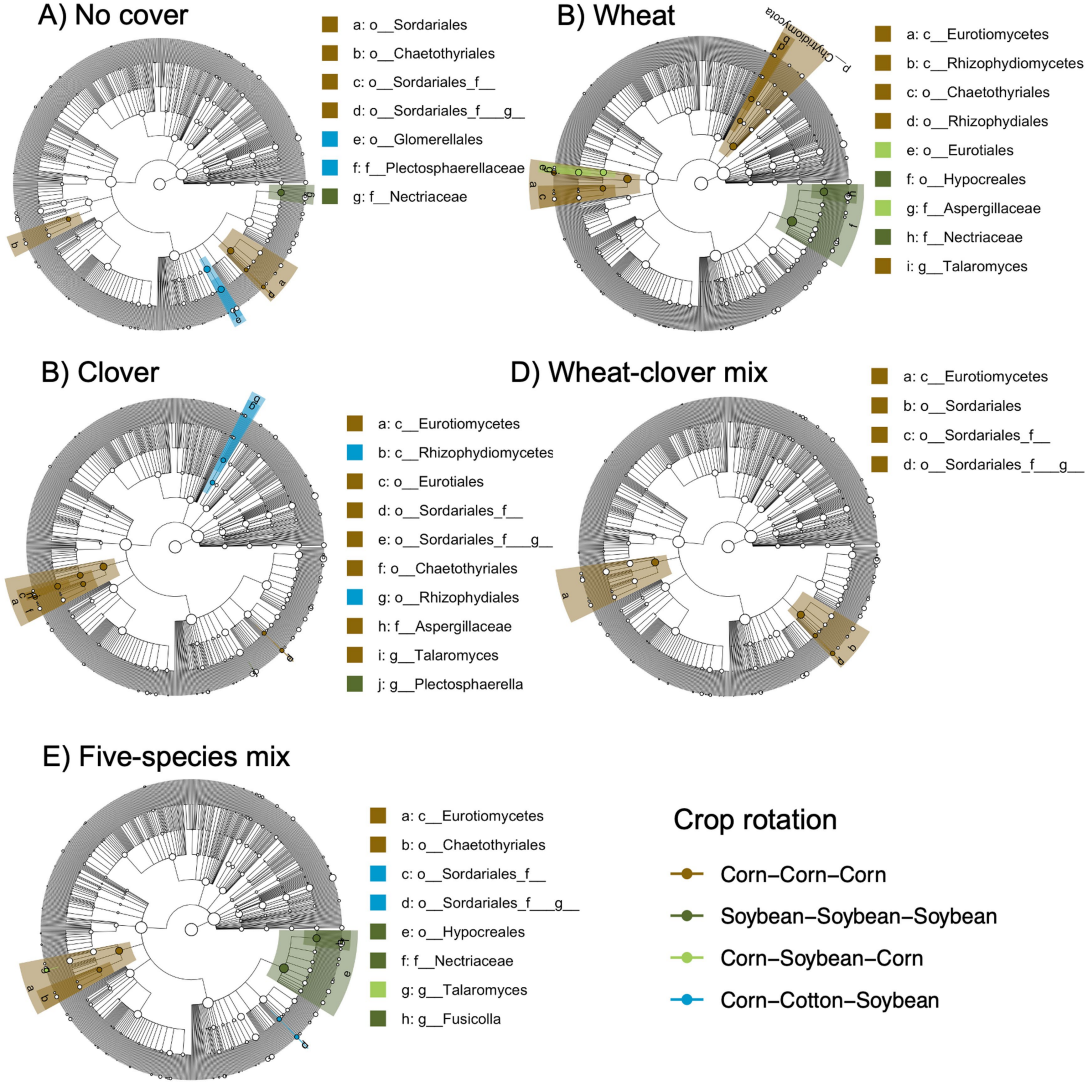
Fungal indicator taxa for different crop rotation treatments across different winter cover crops based on linear discriminant analysis effect size (LEfSE) analysis. Cover crops include **A)** No cover,**B)** Wheat, **C)** Clover, **D)**, Wheat-clover mix, and **E)** Five-species mix. Taxa are mapped onto an ITS-based phylogeny.

Further, fungal community composition showed a two-way interaction between crop rotation and cover crop (Table 2). Additional models indicated that cover crop explained 2.0–2.3% variation (*p* = 0.002) in composition within each crop rotation, and crop rotation explained 4.1–4.4% variation (*p* = 0.002) in composition within each cover crop. Though we did not identify indicator taxa for any cover crop treatment within crop rotations, there were several for crop rotation within cover crops (Figure 5). Notably, the genus *Talaromyces* (saprotroph) was an indicator taxon for continuous corn when the winter cover crop was single-species wheat or clover, and for corn-soybean rotation when the winter cover crop was the five-species mix. The genus *Fusicolla* (mycoparasite) was an indicator taxon for continuous soybean when the winter cover crop was the five-species mix. However, none of the indicator taxa for crop rotations were putative plant pathogens or AMF.

### 3.3 Relationships between soil microbial communities and soil health-related properties

**Figure 6:**
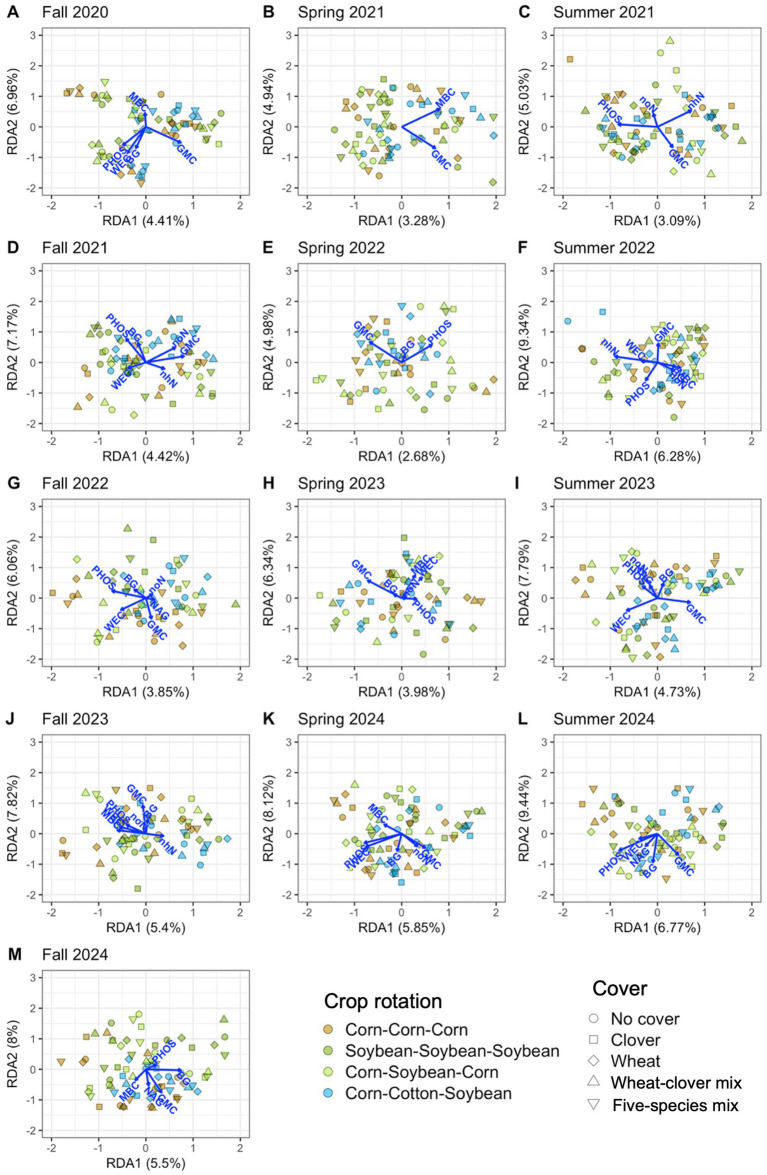
Soil properties associated with bacterial communities from distance-based redundancy analysis. Properties include potential β-glucosidase activity (BG), gravimetric moisture content (GMC), microbial biomass carbon (MBC), potential N-acetyl-β-glucosaminidase activity (NAG), potential phosphatase activity (PHOS), water-extractable organic carbon (WEC), ammonium-nitrogen (nhN), and nitrate-nitrogen (noN). Thirteen timepoints are shown, including fall (A, D, G, J, M), spring (B, E, H, K), and summer (C, F, I, L) in the years 2020 **(A)**, 2021 **(B-D)**, 2022 **(E-G)**, 2023 **(H-J)**, and 2024 **(K-M)**.

**Figure 7:**
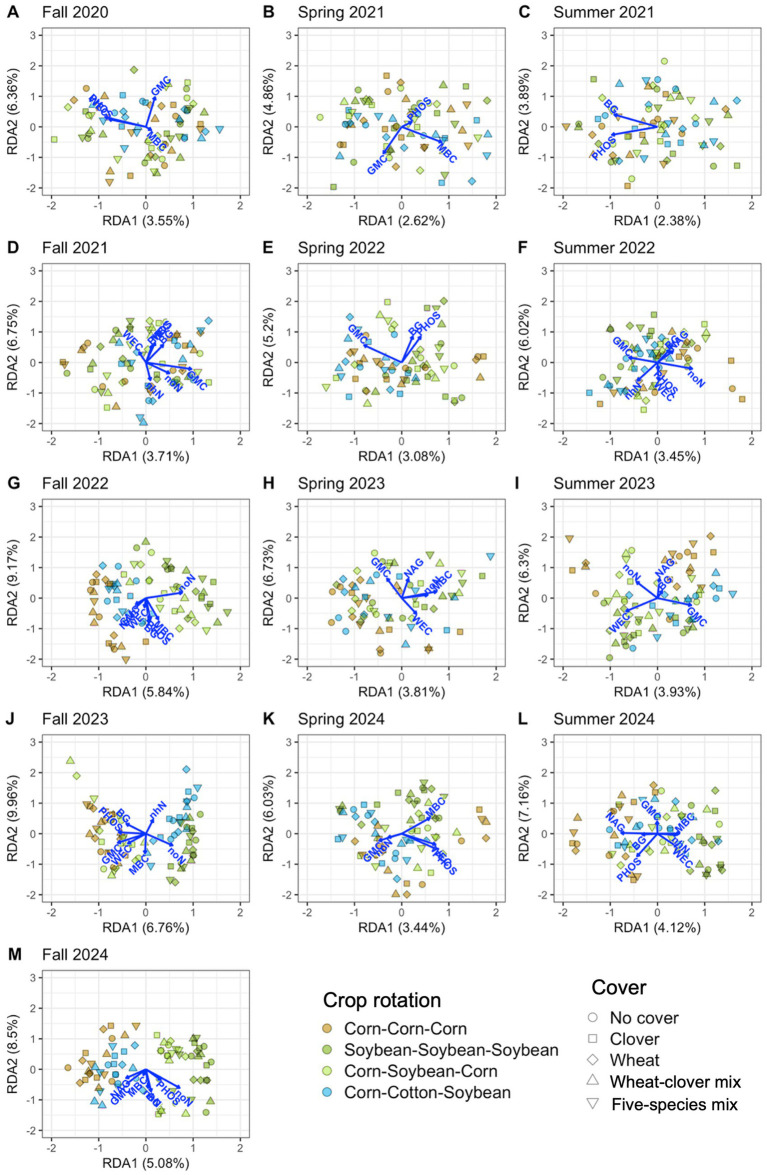
Soil properties associated with fungal communities from distance-based redundancy analysis. Properties include potential β-glucosidase activity (BG), gravimetric moisture content (GMC), microbial biomass carbon (MBC), potential N-acetyl-β-glucosaminidase activity (NAG), potential phosphatase activity (PHOS), water-extractable organic carbon (WEC), ammonium-nitrogen (nhN), and nitrate-nitrogen (noN). Thirteen timepoints are shown, including fall (A, D, G, J, M), spring (B, E, H, K), and summer (C, F, I, L) in the years 2020 **(A)**, 2021 **(B-D)**, 2022 **(E-G)**, 2023 **(H-J)**, and 2024 **(K-M)**.

Soil properties explained significant variation in soil microbial communities at each timepoint, 12.0% on average for bacteria (Table 4 and Figure 6) and 11.9% on average for fungi (Table 4 and Figure 7). Soil moisture appeared as a significant contributor at all timepoints, except for fungi in fall 2022. Bacterial communities were also linked to phosphatase activity (12 timepoints), β-glucosidase activity (11 timepoints), water-extractable organic C (9 timepoints), and nitrate-N (8 timepoints), however each soil property that we measured explained significant variation in bacterial communities in at least four timepoints (Figure 6; Supplementary Figure S2A). Fungal communities were also linked to phosphatase activity (11 timepoints), microbial biomass C, β-glucosidase activity, water-extractable organic C (9 timepoints each), and nitrate-N (8 timepoints) (Figure 7; Supplementary Figure S2B). These results indicate close associations between microbial communities and soil health indicators.

**Table 4:** Total variance in soil microbial community composition explained by soil properties based on distance-based redundancy analysis.

Year	Season	Bacteria	Fungi
2020	Fall (baseline)	11.7%**	9.5%**
2021	Spring	4.9%**	6.4%**
	Summer	7.9%**	5.3%**
	Fall	13.2%**	12.6%**
2022	Spring	6.7%**	8.2%**
	Summer	17.3%**	13.5%**
	Fall	12.9%**	17.7%**
2023	Spring	14.6%**	9.8%**
	Summer	11.7%**	11.3%**
	Fall	14.5%**	17%**
2024	Spring	13.6%**	10%**
	Summer	14.4%**	16.3%**
	Fall	12.4%**	16.7%**

Significance is indicated as ** at *p* < 0.01. Soil properties identified as significant predictors of community composition in each combination of microbial group and timepoint are shown in Figures 6, 7 and Supplementary Figure S2.

## 4 Discussion

### 4.1 Crop diversification does not increase soil microbial diversity

In contrast to our hypothesis that more cropping systems would have higher soil microbial diversity, we found no evidence that crop diversification increases soil bacterial or fungal diversity compared to simplified cropping systems throughout a four-year field experiment. Lack of a strong effect of crop diversification on microbial diversity contradicts results from previous meta-analyses that cover crops increase soil microbial diversity by 3.36% compared to bare fallow (Kim et al., 2020) and crop rotation increases soil microbial diversity by 15.1% compared to continuous cropping (Venter et al., 2016). Still, some field experiments have found no influence or mixed results for crop diversification on soil microbial diversity (Bainard et al., 2020). Those implementing other types of sustainable management practices, such as organic management with cover crops and reduced tillage (vs. conventional fallow and intensive tillage), found limited treatment effects on soil fungal and bacterial diversity (Hartman et al., 2018; Schmidt et al., 2022). Combined with these studies, our results highlight that soil microbial diversity may not always be responsive to changes in agricultural management strategies aimed to enhance soil functioning, as shown in other case studies (Wooliver et al., 2022).

There are three possible reasons why we detected no effects of crop diversification on soil microbial diversity. First, effects of crop diversity on soil microbial diversity may be limited in the short-term and grow more apparent in the long-term. Field experiments 5 years or less in duration showed negligible impacts of crop rotation on soil microbial richness, with experiments six or more years in duration showing positive impacts (Venter et al., 2016). Second, our cover crops produced limited biomass, an average of 1,442 kg ha^−1^ year^−1^ aboveground biomass across all cover-cropped plots and experimental years, which is two to three times lower than average cover crop biomass production in the United States (Blanco-Canqui, 2022). Low cover crop biomass inputs may have limited residue-derived resources important for microbial niche space and subsequent influence on microbial diversity. Third, soil microbial responses to crop diversification may be less evident in bulk soil, where we sampled, compared to rhizosphere soil. In a barley-based cropping system, increased plant diversity through intercropping had strong influence over microbial functioning and community interactions in the rhizosphere (Domeignoz-Horta et al., 2024). Overall, experimental duration, sampling methods, and low cover crop biomass production may all limit soil microbial diversity response to crop diversification.

Microbial functional diversity is increasingly recognized as the link between biodiversity and the functioning of ecosystems (Escalas et al., 2019). Microbial taxa with specific metabolic capabilities, for example N fixation or C mineralization, may be selectively fostered by root exudates or litter inputs from particular crop species. This can lead to a significant change in its collective functioning, better revealed through transcriptomic or metabolomic tools compared to the genomic approach used in our study. For example, in agricultural systems, crop diversity can increase microbial metabolic diversity, determined through the ability of microbial communities to mineralize a variety of C compounds usually present in the rhizosphere (D’Acunto et al., 2018). A meta-analysis of 44 studies showed that crop diversification increases soil microbial N-cycling gene expression (Hao et al., 2022). These studies highlight the importance of assessing microbial function, not just taxon presence and relative abundance, when evaluating soil health in response to land management practices.

### 4.2 Cover crops and crop rotations influence microbial composition

We found mixed support for our hypothesis that the most diverse cropping systems would have the highest abundances of AMF and the lowest abundances of plant pathogens. After the first termination of winter cover crops, the five-species cover crop mix increased the relative abundance of AMF (phylum Glomeromycota, class Glomeromycetes) in soil compared to lower-diversity cover crops and winter fallow. Interestingly, unlike cereal-based systems in which rotation with a legume can increase AMF colonization and yield (Bakhshandeh et al., 2017), we saw no subsequent improvement in cash crop nutrition or yield in plots with the five-species cover crop mix. There were also no effects of cover crops on AMF relative abundances in any season of the three following years. Further, there was no evidence that crop rotation increases AMF relative abundance. Glomeromycota, although representing only 0.5–2% of total fungal abundance (Supplementary Figure S3), constituted over 9% of ASVs in our study, almost twice that detected in European woodlands, grasslands, and croplands (4.9% of ASVs on average) (Labouyrie et al., 2023). Thus, there may have been sufficient AMF diversity in our site to benefit crops regardless of crop diversification strategy. On the other hand, we used herbicides such as glyphosate for cover crop termination and spot-treatment preventing weed growth during the growing season, and these herbicides have been shown to reduce root colonization of AMF (Zaller et al., 2014). It is possible that the AMF taxa identified in our study were a part of the “relic” DNA in the soil, or DNA which is left over from dead microbes (Lennon et al., 2018).

We found strong effects of crop rotations, but not cover crops, on the relative abundances of plant pathogens. In support of our hypothesis, simplified (continuous) cropping systems had higher relative abundances of four fungal plant pathogens. Specifically, continuous corn systems had higher relative abundances of the family Didymellaceae, and continuous soybean had higher relative abundances of the genera *Plectosphaerella*, *Paraphoma*, and *Fusariella*. *Didymella* is a genus within the family Didymellaceae, which is known to cause leaf blight in a range of plant species including corn (Ma et al., 2022), but can affect several other plant organs including roots (Hou et al., 2020). *Plectosphaerella* species have been identified as agents contributing to root rot in several vegetable species (Raimondo and Carlucci, 2018) and a possible agent causing yield decline in continuous soybean (Li et al., 2022). The genus *Paraphoma* contains species known to cause root rot in alfalfa (Cao et al., 2020), and may similarly affect other N-fixing plant species, including soybean. Previous work has shown that despite negligible impacts of crop diversity on soil microbial diversity, influence on soil microbial community composition was linked to disease suppressive function, lessening vulnerability to disease in diverse crop rotation compared to monoculture corn (Peralta et al., 2018). We did find that rotation significantly increased corn and soybean yield in the first 3 years of the experiment. Further, in the fourth year, rotation of corn with cotton and soybean increased yield by 21.2% compared to continuous corn (Supplementary Figure S4). Because we did not see any visual signs of leaf or grain infection in continuous cropping systems, we speculate that root pathogens may have played a bigger role in contributing to yield effects of crop rotation, but more work is required to support this prediction. Further, the lack of cover crop effects on plant pathogen abundances indicates that they did not serve as a green bridge for pathogen transfer between annual cash crops in our study, in contrast to the green bridge effect seen elsewhere (Acharya et al., 2017). We allowed at least 2 weeks after cover crop termination to initiate cash crop planting, which might have helped to reduce transfer of plant pathogens in our study.

Despite mixed results for the effects of crop diversification on AMF and plant pathogens, we found strong effects on other major microbial groups and on overall community composition. Fungal community composition was more responsive to crop diversification compared to bacterial community composition. Further, crop rotation had stronger influences on both microbial groups compared to cover crop diversity. Similarly, a field experiment in China found that crop rotation in soybean- and corn-based systems had strong influences on fungal and bacterial communities (Sun et al., 2023). We found that Actinobacteria were relatively more abundant in continuous corn compared to all other cropping systems. Actinobacteria grow and reproduce quickly in an abundance of resources (Stone et al., 2023), which may be a result of high chemical N fertilization rates provided to corn compared to cotton and soybean. Alternatively, Acidobacteria were relatively more abundant in continuous soybean, which may be attributed to their preference for C-limited environments (Fierer et al., 2007) and lower overall biomass production from soybean compared to corn. The fungal genus *Talaromyces* was relatively more abundant in continuous corn and corn-soybean systems. *Talaromyces* has been recognized as an important decomposer in agroecosystems, contributing to both soil health and disease resistance (Abbas et al., 2024). A separate field experiment manipulating other management strategies, specifically tillage intensity and conventional vs. organic management, found that these practices explained about 10% of variation in soil fungal and bacterial communities (Hartman et al., 2018). Compared to our estimates (Table 3), this suggests that crop rotation can have equal or greater influence over soil microbial communities compared to other agricultural management strategies, while cover cropping has a lesser influence.

Cover crop effects on soil microbial communities could have been limited by poor cover crop establishment and biomass production, as discussed in the previous section. As opposed to crop rotation whose impacts on both bacterial and fungal community composition grew over time, cover crop treatment was more likely to impact fungal community composition in the spring (after cover crop termination) and summer (during cover crop residue decomposition) compared to the fall (after most of the cover crop residues had likely decomposed). This indicates that cover crop effects on fungal communities are time-dependent, which has implications for conclusions drawn from studies that collect soils in only one season or year.

### 4.3 Microbial community composition is linked to soil health

In support of our hypothesis that microbial communities would be associated with soil health indicators, we found tight linkages between soil microbial communities and moisture content, enzyme activities, microbial biomass C, inorganic N, and water-extractable organic C. These properties, combined, explained about one-tenth of variation in microbial community composition across timepoints (Table 4). Notably, only towards the end of the four-year experiment did crop rotations more clearly shape microbial (especially fungal) community composition and associated soil properties (Figures 6, 7). However, additional exploration of data from our experiment suggested that effects of crop diversification on soil health were limited (Supplementary Tables S2, S3 and Supplementary Figures S5–S7), suggesting that crop diversification can shape microbial communities independent of changes in soil health.

Notably, no soil properties were related to the increase in the relative abundance of AMF by five species cover crop mix in the spring of 2021. Although pot experiments have shown that AMF inoculation significantly increases soil organic C by an average of 40%, field studies result in increases of only 11% (non-significant) (Conti et al., 2025). Combined, these results suggest that increasing AMF abundance through crop diversification may not be an effective pathway to increase soil health in the field short-term (after one cover crop season). Further, we expected C content of corn residues to induce N- and P-limitation in soil microbes, and for this to alter saprotroph communities and enzyme activity in continuous corn-based systems. Consistent with this prediction, we identified *Talaromyces*, a genus which has a high capacity for nutrient mineralization (Kanse et al., 2015), as an indicator taxon for corn-based systems in our study. However, potential enzyme activities were not higher in our corn-based systems (Supplementary Figure S5). Additionally, Actinobacteria are known to prefer mesophilic conditions (Barka et al., 2015) and this group was an indicator taxon for corn-based systems, however we did not see significantly higher soil moisture under corn (Supplementary Figure S6). As opposed to nutrient limitation, enzyme activities, and moisture, inorganic N levels modulated by soybean appeared to be more strongly linked to both crop diversification and microbial community composition. Soybean phases of continuous soybean and corn-soybean rotation had the highest soil nitrate-N concentrations across the fall timepoints of 2022–2024 (Supplementary Figure S7), which may have contributed to the increased abundances of Acidobacteria and the saprotroph *Ramicandelaber* in these cropping systems. Very little is known about the ecology of *Ramicandelaber*, however Acidobacteria are known to be nitrate reducers (Kielak et al., 2016). Ultimately, differences in crop rotations culminated in highly distinct communities during fall 2024, where bacterial and especially fungal microbial communities were similar between corn-cotton-soybean and continuous corn, as opposed to corn-soybean rotation and continuous soybean (Figures 6, 7). This indicates the growing influence of crop rotation, and especially the incorporation of N-fixers such as soybean into crop rotations, on soil microbial communities over time.

## 5 Conclusion

Four years of crop diversification via winter cover crops and row crop rotation revealed limited effects on soil microbial diversity in a western Tennessee silt loam soil, but clear influences over soil bacterial and fungal communities. Our results indicated that crop rotation has stronger effects on bacterial and fungal communities compared to cover cropping, regardless of season or year. Further, we found that fungal communities respond more strongly to crop rotation than bacteria. Notably, rotations decreased relative abundances of fungal taxa known to be plant pathogens compared to simplified continuous corn and soybean systems, supporting the wealth of literature showing that fungal pathogens mediate rotation-driven yield increases. Further, soils collected in the fall showed strong bacterial taxa-specific responses to monocropped corn or soybean, suggesting that bacterial communities are sensitive to decomposing crop-specific residues. We found that cover crops, on the other hand, do not influence bacterial communities and have ephemeral effects on fungal communities, more likely to shift overall communities during active cover crop residue decomposition (spring > summer > fall). A diverse cover crop mix increased relative abundances of the AMF-dominant phylum Glomeromycota in the spring of only the first experimental year, suggesting that early microbial responses to cover crops may not be indicative of longer-term responses. Finally, our study showed strong linkages between microbial communities and soil health indicators, despite minimal effects of crop diversification on soil health indicators. Overall, we conclude that crop rotation and cover cropping have strong, but separate, potentials to influence the abundances of fungal pathogens and AMF, largely independently of changes in soil health.

## Data Availability

The datasets presented in this study can be found in online repositories. The names of the repository/repositories and accession number(s) can be found at: https://www.ncbi.nlm.nih.gov/, PRJNA1265188.
