# Supplementary material for: Microbial communities and their association with soil health indicators under row cash crop and cover crop diversification: a case study
Source: Front Microbiol. 2025 Sep 29;16:1664417. doi: 10.3389/fmicb.2025.1664417 (PMC12515911; doi:10.3389/fmicb.2025.1664417)

Supplementary material for:

Microbial communities and their association with soil health indicators under row cash crop and cover crop diversification: A case study

Table S1: Three-way ANOVA of cover crop, crop rotation, and timepoint effects on microbial diversity indices. Values represent F-values. Significant treatment effects at P<0.05, P<0.01, and P<0.001 are marked with *, **, and ***, respectively.

	Bacteria				Fungi			
	Shannon diversity	Simpson diversity	Inverse Simpson diversity	Evenness	Shannon diversity	Simpson diversity	Inverse Simpson diversity	Evenness
Cover	0.16	0.16	0.45	0.39	0.06	0.07	0.22	0.07
Rotation	0.56	0.12	0.62	0.63	0.16	0.08	0.42	0.12
Timepoint	4.66 ***	0.64	2.48 **	0.81	1.61	0.81	1.21	1.26
Cover x Rotation	1.06	0.79	1.06	0.8	0.2	0.13	0.27	0.12
Cover x Timepoint	0.57	0.34	0.52	0.7	0.74	0.95	0.82	0.78
Rotation x Timepoint	0.66	0.53	0.76	0.79	1.25	0.66	1.17	1.16
Cover x Rotation x Timepoint	0.7	1.14	0.89	1.07	0.82	0.8	0.88	0.84

Table S2: Three-way ANOVA of cover crop, crop rotation, and timepoint effects on soil properties. Values represent F-values. Properties include potential β-glucosidase activity (BG), gravimetric moisture content (GMC), microbial biomass carbon (MBC), potential N-acetyl-β-glucosaminidase activity (NAG), potential phosphatase activity (PHOS), water-extractable organic carbon (WEC), ammonium-nitrogen (NH_4^+-N), and nitrate-nitrogen (NO_3^--N). Significant treatment effects at P<0.05, P<0.01, and P<0.001 are marked with *, **, and ***, respectively.

	BG	GMC	MBC	NAG	NH4+-N	NO3--N	PHOS	WEC
Cover	0.57	0.35	0.58	0.18	0.22	0.93	0.71	0.1
Rotation	0.42	0.83	1.46	0.15	0.33	0.83	0.4	0.16
Timepoint	2.39 **	3.7 ***	3.55 ***	1.6	2.65 **	6.74 ***	2.94 **	4.46 ***
Cover x Rotation	0.44	0.58	1.86 *	0.18	0.21	1.71	0.52	0.61
Cover x Timepoint	0.38	0.39	0.41	0.9	1.57 **	0.67	0.48	0.59
Rotation x Timepoint	0.62	0.92	0.6	0.85	0.66	1.96 **	0.81	0.87
Cover x Rotation x Timepoint	0.5	0.38	0.46	0.62	0.86	0.66	0.57	0.51

Table S3: Two-way ANOVA of cover crop and crop rotation effects on soil properties across different sampling points. Values represent F-values. Properties include ammonium-nitrogen (NH_4^+-N) and nitrate-nitrogen (NO_3^--N). Significant treatment effects at P<0.05, P<0.01, and P<0.001 are marked with *, **, and ***, respectively.

		2021			2022			2023			2024		
		Spring	Summer	Fall	Spring	Summer	Fall	Spring	Summer	Fall	Spring	Summer	Fall
NH_4^+-N	Cover	0.16	0.24	0.97	5.53 **	0.64	0.94	0.70	0.21	1.21	0.38	0.14	0.76
	Rotation	4.53 **	1.08	2.16	3.69 *	0.41	0.35	0.58	2.16	0.56	0.95	0.97	1.40
	Cover x Rotation	1.8	0.65	1.30	1.37	0.53	0.68	0.94	0.64	0.92	1.15	0.88	0.75
NO_3^--N	Cover	4.15 **	1.33	0.38	6.36 ***	0.11	0.35	3.02 *	7.29 ***	0.79	3.00 *	0.82	0.33
	Rotation	0.38	0.23	0.76	0.45	0.54	12.25 ***	0.63	0.58	6.36 **	0.87	1.16	4.75 **
	Cover x Rotation	1.86	0.93	0.78	0.88	0.87	0.50	0.57	1.56	1.88	1.23	0.93	0.35

Figure S1: Bacterial and fungal richness across time for each cover crop treatment.

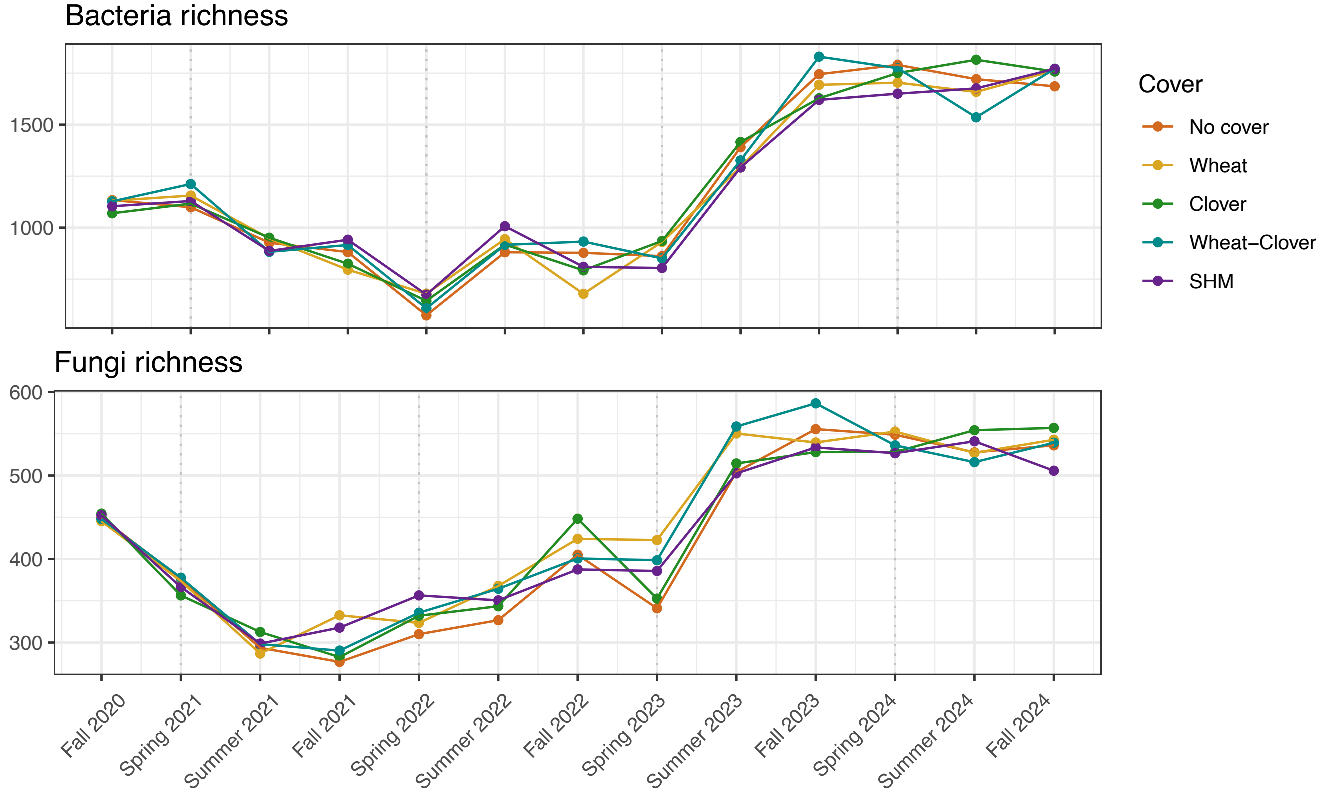

Figure S2: Bacterial Shannon and Inverse Simpson diversity across time for each cover crop treatment.

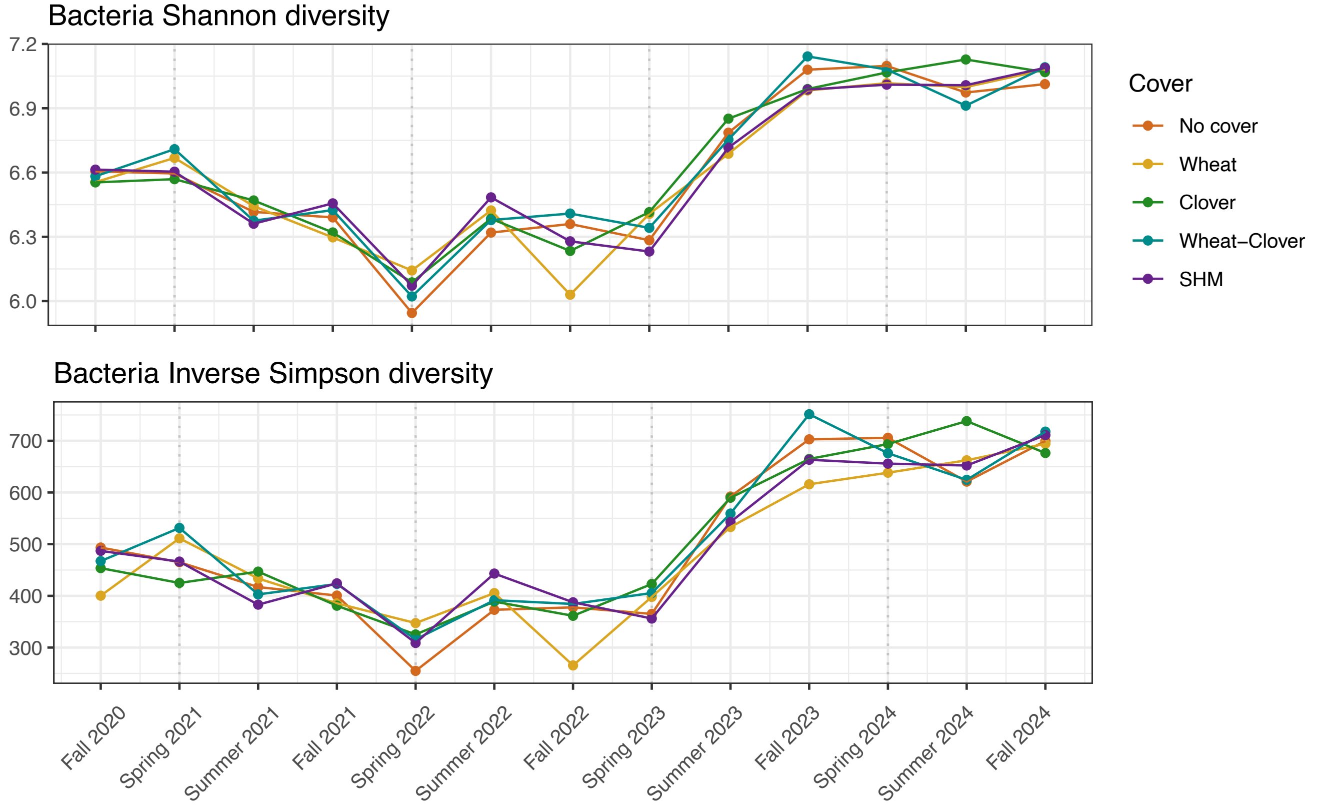

Figure S2: Variance explained in microbial community composition by each soil health-related property across timepoints based on distance-based redundancy analysis. Properties include potential β-glucosidase activity (BG), gravimetric moisture content (GMC), microbial biomass carbon (MBC), potential N-acetyl-β-glucosaminidase activity (NAG), potential phosphatase activity (PHOS), water-extractable organic carbon (WEC), ammonium-nitrogen (nhN), and nitrate-nitrogen (noN).

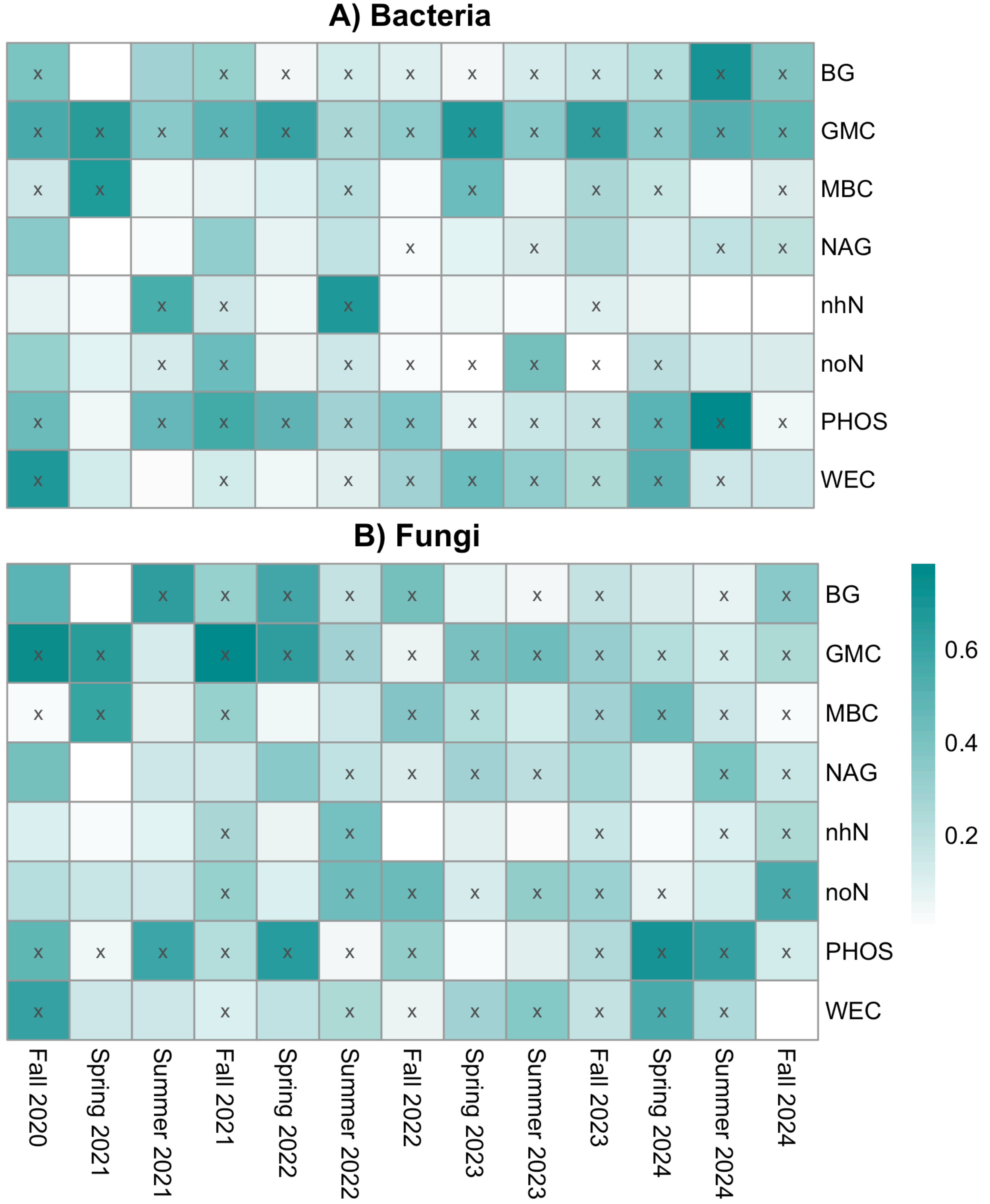

Figure S3: Relative abundance of the 10 most abundant fungal phyla, averaged across all samples within each timepoint.

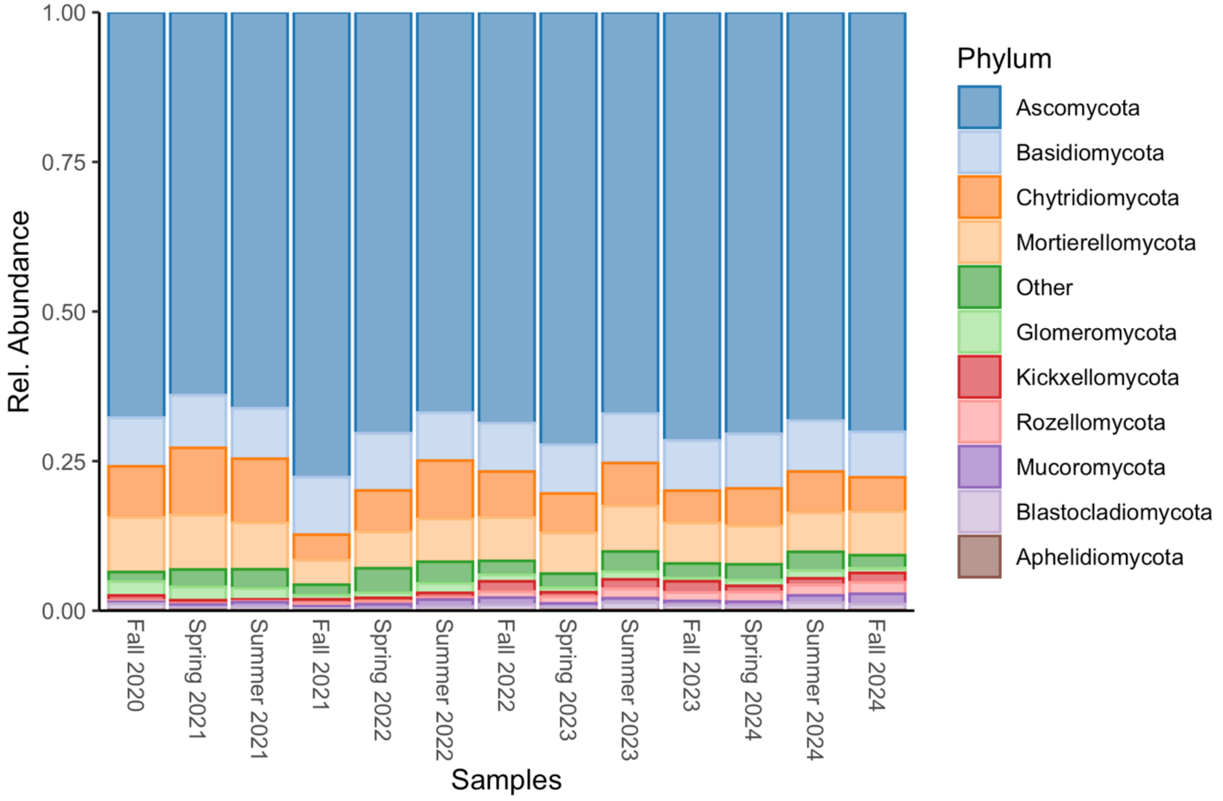

Figure S4: Corn yield in 2024. Error bars represent standard error, and letters represent the difference between cropping systems according to Tukey's post-hoc test.

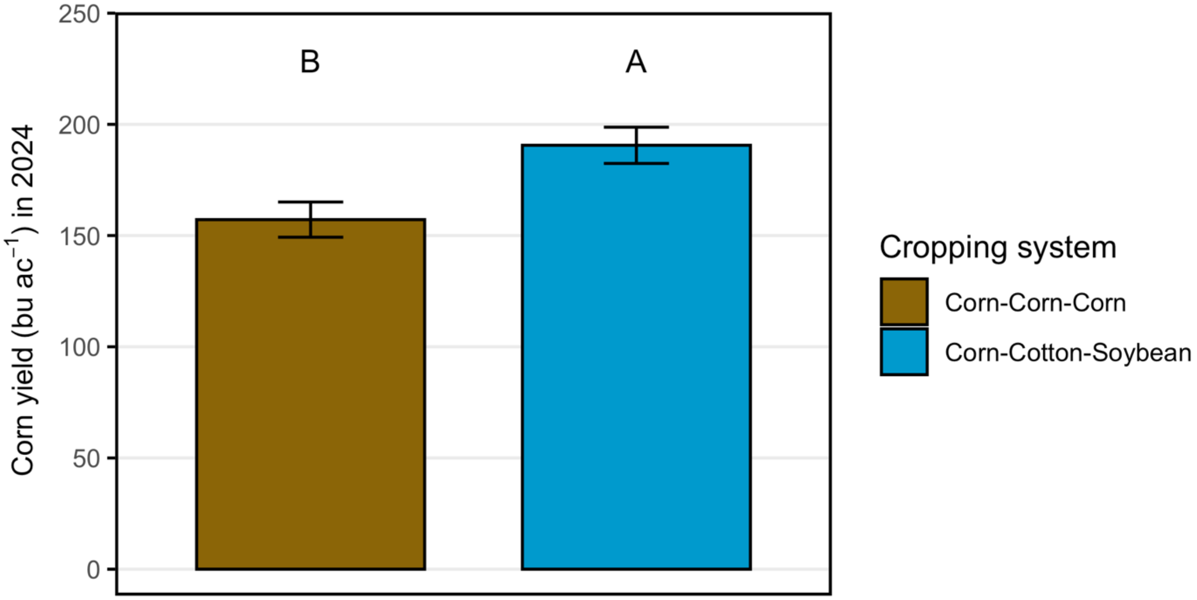

Figure S5: Potential enzyme activities across time for each crop rotation treatment.

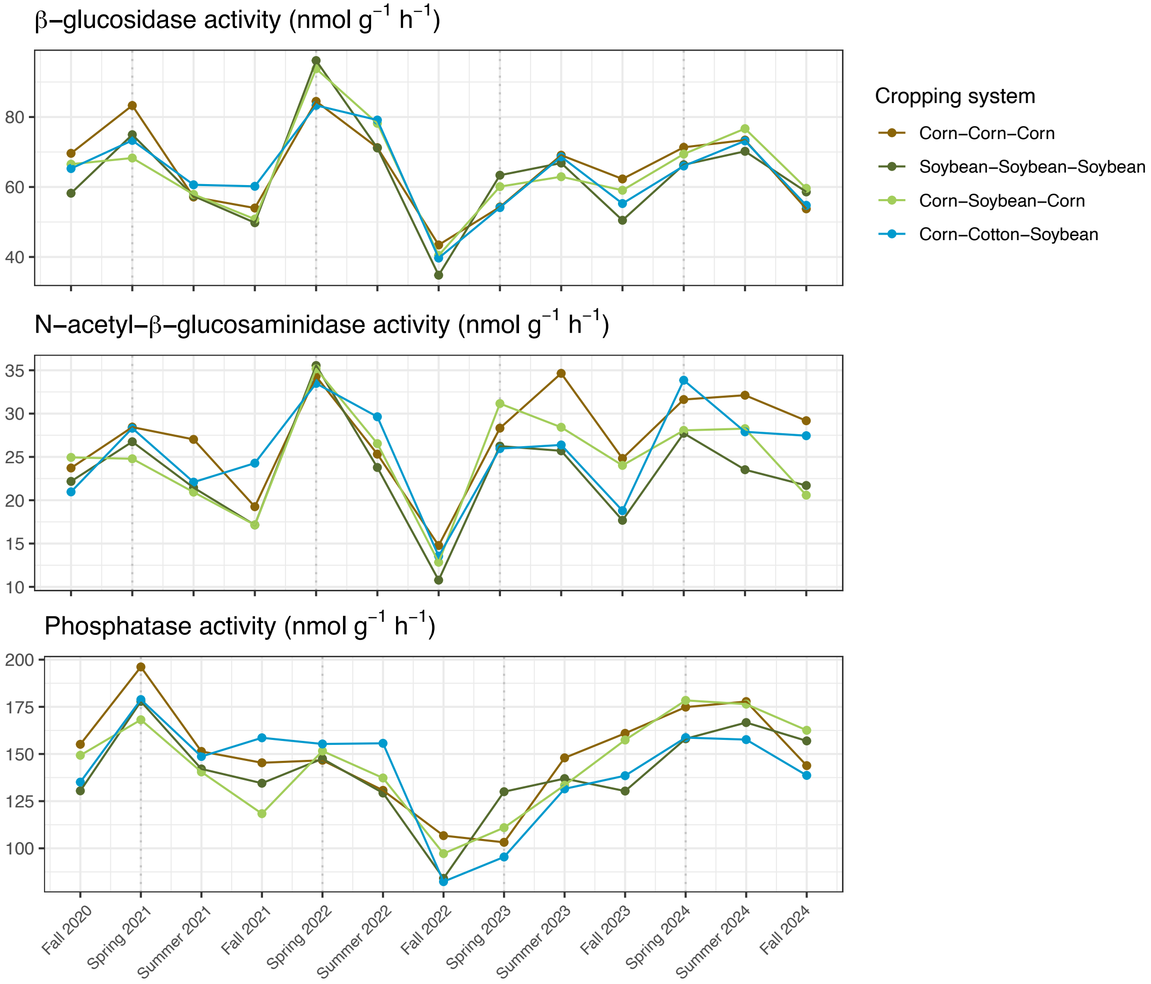

Figure S6: Gravimetric moisture content and microbial biomass carbon across time for each crop rotation treatment.

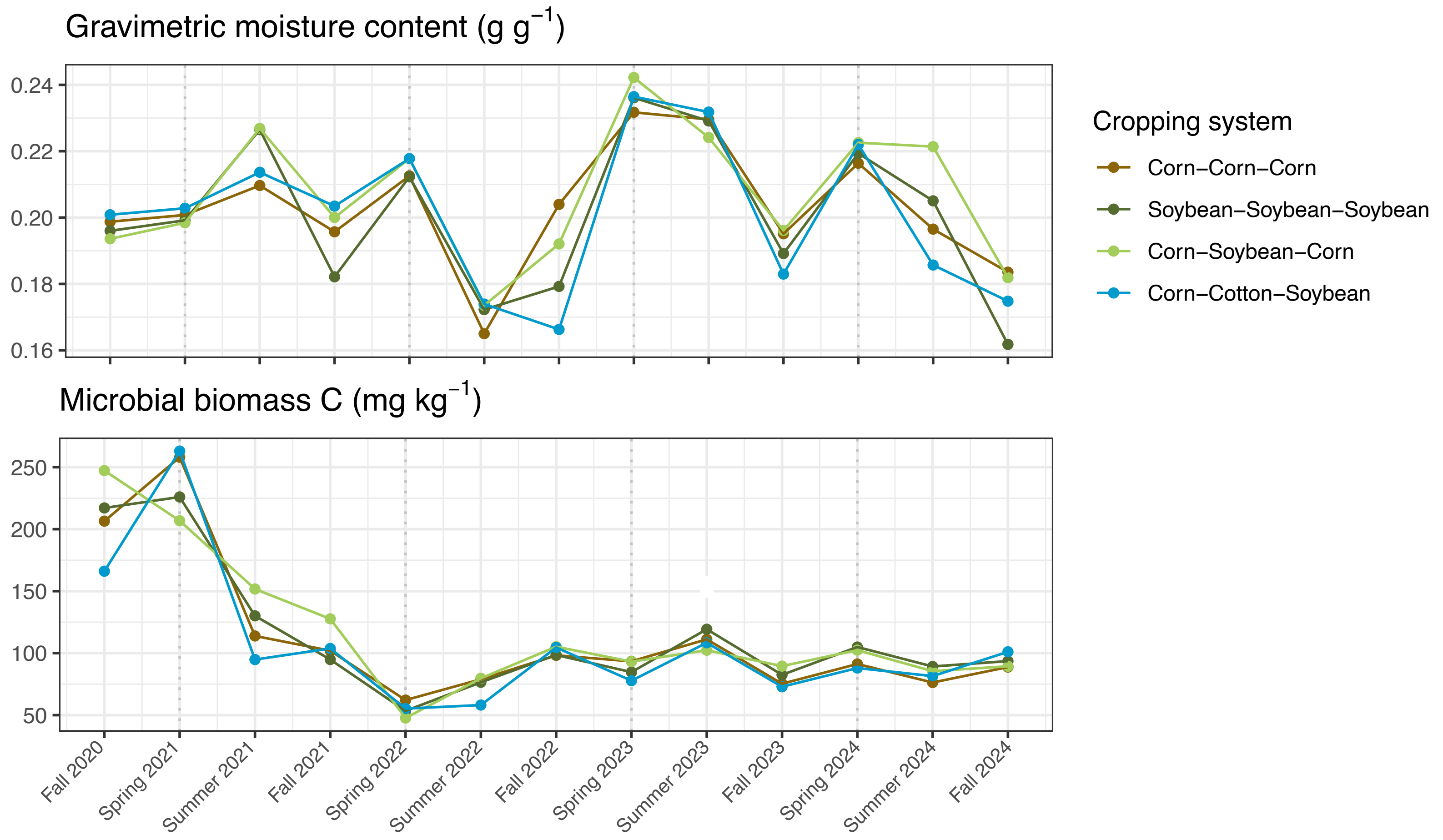

Figure S7: Water-extractable organic carbon, ammonium-nitrogen, and nitrate-nitrogen across time for each crop rotation treatment.

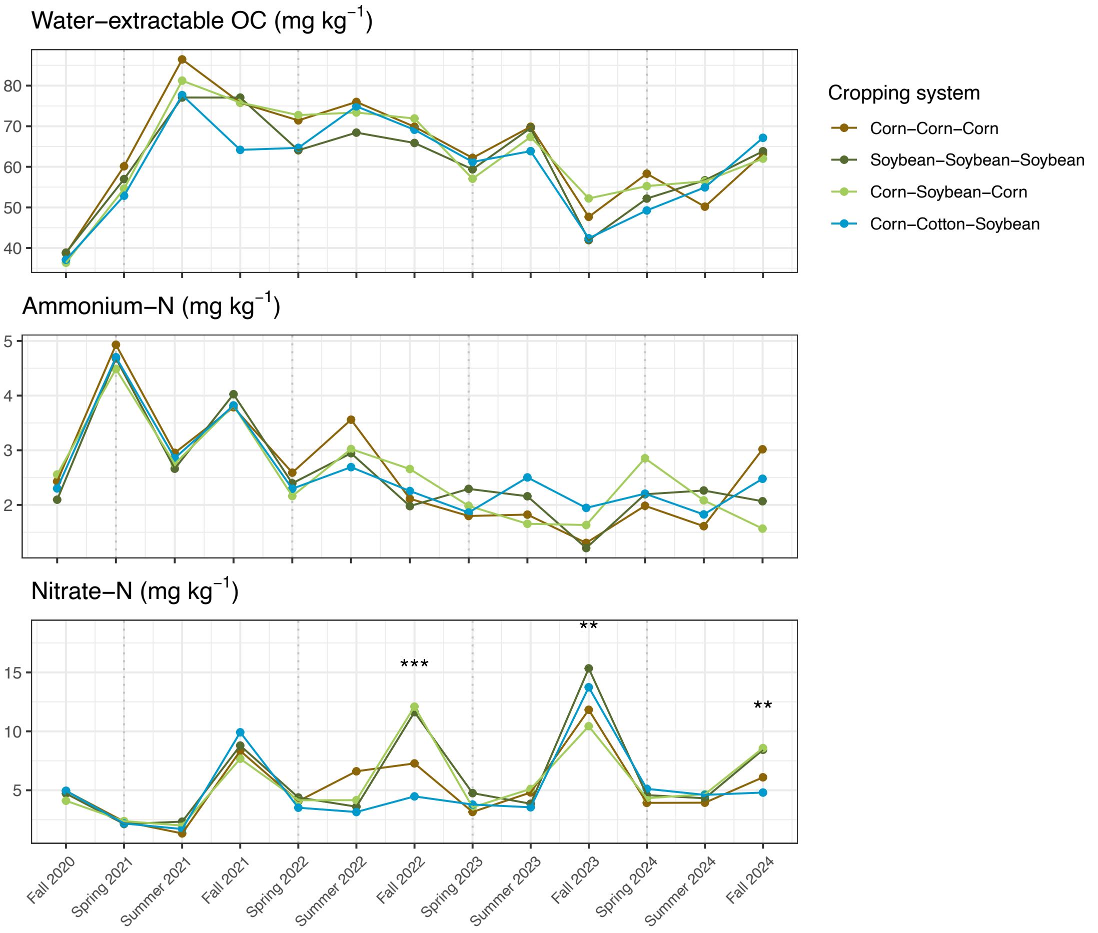

Supplement: Supplementary file 1 [file Data_Sheet_1.pdf]
